# Metabolism and immune responses of striped hamsters to ectoparasite challenges: insights from transcriptomic analysis

**DOI:** 10.3389/fimmu.2024.1516382

**Published:** 2024-12-12

**Authors:** Xinchang Lun, Yiguan Wang, Ning Zhao, Yujuan Yue, Fengxia Meng, Qiyong Liu, Xiuping Song, Ying Liang, Liang Lu

**Affiliations:** ^1^ National Key Laboratory of Intelligent Tracking and Forecasting for Infectious Diseases, National Institute for Communicable Disease Control and Prevention, Chinese Center for Disease Control and Prevention, Beijing, China; ^2^ CAS Key Laboratory of Insect Developmental and Evolutionary Biology, CAS Center for Excellence in Molecular Plant Sciences, Shanghai, China

**Keywords:** ectoparasites parasitism, striped hamsters, transcriptome analysis, metabolism, immunity

## Abstract

**Introduction:**

The striped hamster, often parasitized by ectoparasites in nature, is an ideal model for studying host-ectoparasite molecular interactions. Investigating the response to ectoparasites under laboratory conditions helps elucidate the mechanism of host adaptations to ectoparasite pressure.

**Methods:**

Using transcriptome sequencing, we analyzed gene expression in striped hamsters after short-term (3 days) and long-term (28 days) flea (*Xenopsylla cheopis*) parasitism. Differentially expressed genes (DEGs) were identified and subjected to Gene Ontology (GO) and Kyoto Encyclopedia of Genes and Genomes (KEGG) enrichment analyses. Hub genes were pinpointed using protein-protein interaction (PPI) network analysis and the MCODE in Cytoscape. Gene Set Enrichment Analysis (GSEA) was used to further clarify the functional pathways of these hub genes. Validation of DEGs was performed via RT-qPCR. Additionally, the concentrations of reactive oxygen species (ROS), superoxide dismutase (SOD), glutathione peroxidase (GSH-Px) and catalase (CAT) were determined using specific enzyme-linked immunosorbent assay (ELISA) detection kits for hamsters.

**Results:**

GO analysis revealed that during early parasitism, hosts primarily responded to the ectoparasites by adjusting the expression of genes related to metabolic functions. As parasitism persisted, the immune response became prominent, activating various immune pathways against ectoparasites. KEGG analysis confirmed the ongoing roles of metabolism and immunity. Notably, the chemical carcinogenesis - reactive oxygen species pathway was upregulated during flea parasitism, with downregulation of hub genes ATP5MC1 and ATP5MC2, highlighting the importance of mitochondrial function in oxidative stress. ELISA findings revealed that on day 3, flea parasitism groups showed elevated ROS expression and reduced SOD and CAT levels compared to the control group. By day 28, only SOD expression showed a significant decrease in both parasitism groups.

**Conclusion:**

This study uncovered the dynamic changes in metabolism and immune responses of striped hamsters parasitized by *Xenopsylla cheopis*. Hosts adjust their physiological and immune states to optimize survival strategies during different ectoparasite stages, enhancing our understanding of host-ectoparasite interactions. This also paves the way for further research into how hosts regulate complex biological processes in response to ectoparasite challenges.

## 1 Introduction

Striped hamsters are small rodents widely distributed in East Asia (1), with high reproductive capacity (2, 3). They play a crucial role in maintaining ecosystem balance and biodiversity (4), but also significantly impact disease transmission and lead to economic loss in agriculture. Fleas commonly infest hamsters (5), capable of spreading various pathogens. Among them, the *Xenopsylla cheopis* (*X. cheopis*) not only poses a direct threat to striped hamsters but also has the potential to spread various pathogens (6, 7) to humans.

The parasitic behavior of fleas extend beyond physical irritation, triggering complex immune responses, inflammation and physiological adjustments in the host (8), including changes in temperature regulation, energy metabolism and behavioral patterns (9, 10). These reactions can lead to changes in gene expression related to immune response, inflammation regulation and neural signaling. Hosts may adapt by modulating gene expression to enhance defense mechanisms or mitigate discomfort (11). It is worth noting that the salivary glands of *X. cheopis* contain various proteins and peptides which not only help *X. cheopis* complete the blood-sucking process but also inhibit the host’s immune response (12). For example, thrombin inhibitors like XC-42 and XC-43 disrupt coagulation, aiding continuous feeding (13, 14), while the FS family peptide uniquely impacts the function of the host immune system (15–18).

The intricate interplay between fleas and their hosts offers a window into understanding the adaptive evolutionary response of hosts facing persistent ectoparasite challenges. Striped hamster, is highly sensitive to pathogens and the wild population tends to have a high rate of flea infection. In addition, some unique characteristics [e.g., small body size, large testicles, long lifespan and low chromosome count (19)] enables the striped hamster to be an ideal experimental model for studying pathogenic infections.

Previous studies has largely focused on specific immunoglobulin expressions or inflammatory cell responses post-*X. cheopis* parasitization using techniques like flow cytometry, histopathology and antibody detection (20–22). Some studies have explored the immune-related regulatory mechanisms that occurred in host cells activated by ectoparasite saliva components (23, 24). However, transcriptome sequencing represents a more holistic methodology, enabling a deep dive into the comprehensive gene expression shifts in hosts following ectoparasites exposure. By RNA sequencing (RNA-seq), the composition and expression levels of transcripts can be determined, helping to reveal the molecular mechanisms of gene expression differences between individuals and related genes (25–28). In this study, we employed transcriptome sequencing to map the temporal dynamics of gene expression changes in striped hamsters subjected to varying durations of flea parasitism. The objective is to construct a detailed picture of gene expression changes and further explore the potential immune response pathways and regulatory mechanisms of hosts after experiencing ectoparasitic infections.

## 2 Methods

### 2.1 Ethics statement

All animal experiments were conducted in accordance with the National Health guidelines for the welfare of experimental animals and with the approval of the Ethical Committee of the National Institute for Communicable Disease Control and Prevention, Chinese Center for Disease Control and Prevention.

### 2.2 Animals and sample collection

All 36 clean-grade male striped hamsters used in the experiment (License Number: SCXK (Jin) 2019-0004) were purchased from the Experimental Animal Center of Shanxi Medical University. These hamsters were randomly divided into 3 groups, with 12 hamsters in each group and each hamster was kept in a single cage. The first group served as a blank control group without exposure to *X. cheopis*; The second group was the low-intensity flea parasitism group, with 20 *X. cheopis* placed on each striped hamster; The third group served as a high-intensity flea parasitism group, with 50 *X. cheopis* placed on each striped hamster. Before the experiment, the key basic information such as the weight and food supply of each hamster was recorded. On the 3rd, 7th, 14th and 28th days, the striped hamsters were anesthetized and euthanized using inhalation of an overdose of isoflurane, ensuring a humane and painless end. Their weight, remaining food intake, body fleas and nest fleas were recorded. Then, the hamsters were quickly dissected and liver samples were taken. Liver samples from hamsters on day 3 and day 28 were selected for transcriptome analysis.

*X. cheopis* were initially collected from the *Rattus norvegicus* in the suburban district of Siping City, Jilin Province (Northeast China) in 2002, and were then introduced to our laboratory in 2003. The population of fleas has been maintained in laboratory conditions since then on specific pathogen-free (SPF) mice. The mice were placed in a cage measuring approximately 10 cm in length and 4.5 cm in diameter, which was then positioned inside a culture dish lined with filter paper. Following this, a 30 cm × 30 cm glass jar was preheated by baking for about two hours. After cooling to room temperature, the jar was filled with 1-2 cm of feed mixture (composed of pig blood powder, yeast powder and fine sand in a 1:1:10 ratio). The culture dish containing the mice was then centered within the jar, and adult fleas were introduced to feed on the mice’s blood. The eggs on the filter paper were collected and placed in a culture dish containing feed until they formed cocoons. Cocoon pupae were screened and stored in test tubes or collected from fleas for experimentation.

### 2.3 RNA extraction

Total RNA was extracted from 18 liver samples of striped hamsters from the control group, low-intensity flea parasitism group and high-intensity flea parasitism group on days 3 and 28 using the TRIzol method. An equal amount of liver samples were collected for each group to ensure comparability and reliability of the data. Nanodrop was used to measure the concentration of RNA. Agilent 2100 Bioanalyzer was used to accurately examine the integrity of RNA to achieve strict quality control of extracted RNA.

### 2.4 Library construction and sequencing

The sequencing library was constructed as follows: mRNA with polyA tail was enriched by magnetic beads containing Oligo (dT), and the obtained mRNA was randomly broken into short fragments by divalent cations in Fragmentation Buffer. Using interrupted mRNA as a template and random oligonucleotides as primers, the first strand of cDNA was synthesized in the M-MuLV reverse transcriptase system. Subsequently, RNA strands were degraded by RNaseH, and the second strand of cDNA was synthesized by dNTPs as raw material in the DNA polymerase I system. After synthesizing cDNA strands, the cDNA was purified, and the purified double-stranded cDNA was subjected to end repair, followed by adding an a-tail and connecting to a sequencing adapter. Then, AMPure XP beads were used to screen out cDNA with a length of approximately 370-420 bp. PCR amplification was performed, and the PCR product was purified again using AMPure XP beads, ultimately the cDNA library was obtained.

Quality control was performed on the constructed library. First, Qubit 2.0 Fluorometer was used to perform preliminary quantification on each library diluted to 1.5 ng/ul. Then Agilent 2100 Bioanalyzer was used to examine the insert size of the library. Then qRT-PCR was used to accurately quantify the effective concentration of the library.

After the quality control of the library, Illumina sequencing was performed to generate 150 bp paired-end reads, targeting a data volume of 6GB for each sample.

### 2.5 Bioinformatics analysis

#### 2.5.1 Data processing and transcriptome assembly

For the raw data in fastq format, fastp (29) was used to filter out sequencing adapters and low-quality reads (i.e., reads with over 50% bases having a phred-score less than 5) to obtain clean reads. HISAT2 (30) was used to map clean reads to the reference genome of *Cricetulus griseus* (ncbi_cricetulus_griseus_gcf_000223135_1_crigri_1_0). FeatureCounts (31) was used to quantify the reads mapped to each gene. Finally, FPKM (Fragments Per Kilobase of exon model per Million mapped fragments) was used to correct for the effects of sequencing depth and gene length.

#### 2.5.2 Analysis of differentially expressed genes

DESeq2 (32) was used for comparative analysis of the obtained gene expression levels, including the following four comparison groups: the day 3 low-intensity flea parasitism group vs. control group; the day 3 high-intensity group vs. low-intensity group; the day 28 low-intensity group vs. control group; and the day 28 high-intensity group vs. low-intensity group. In the comparison of each group, the fold-change and significance of gene expression levels were calculated, and a *P*-value < 0.05 was selected as the threshold, simultaneously ensuring that the expression level of a certain gene in all samples within a certain group was higher or lower than that of all samples within another group. Based on these criteria, we identified significant DEGs and ultimately obtained four groups of DEGs.

#### 2.5.3 Gene Ontology and Kyoto Encyclopedia of Genes and Genomes enrichment analysis

Firstly, four sets of common trend (the pattern of DEGs that showed a consistent direction of change across different conditions) DEGs were obtained. These four comparison groups included: Early-stage intensity-dependent DEGs (genes with consistent expression changes between high-intensity group vs. low-intensity group and low-intensity group vs. control group at 3 days); Late-stage intensity-dependent DEGs (genes with consistent expression changes between high-intensity group vs. low-intensity group and low-intensity group vs. control group at 28 days); Low-intensity time-dependent DEGs (genes with consistent expression trends between day 3 and day 28 under low-intensity group vs. control group); High-intensity time-dependent DEGs (genes with consistent expression trends between day 3 and day 28 under high-intensity group vs. low-intensity group.

ClusterProfiler (33) was used to perform GO term and KEGG pathway enrichment analysis of common trend DEGs. *P*-value < 0.05 was used as the threshold for significant enrichment in GO and KEGG analysis. The emapplot network diagram was used to cluster similar terms of significantly enriched GO terms together to identify different GO functional modules. The treeplot function was used to display terms that approximate the significantly enriched KEGG pathways in the form of clustering trees, which facilitates the identification of different KEGG functional modules. All of these analyses were conducted in R software.

#### 2.5.4 Protein-protein interaction network analysis

We summarized DEGs related to the metabolism and immune function of the host through GO and KEGG enrichment analysis. The STRING database (http://string-db.org) was used to analyze the PPI network of DEGs and the Cytoscape software (34) was used to visualize the PPI network of DEGs. The “MCODE” plugin from Cytoscape was used to identify the most important clustering modules in PPI networks, where genes within the clustering modules were typically considered key hub genes.

#### 2.5.6 Gene set enrichment analysis

The clusterProfiler was used to perform GSEA analysis on all annotated DEGs in four groups: the day 3 low-intensity group vs. control group; the day 3 high-intensity group vs. low-intensity group; the day 28 low-intensity group vs. control group and the day 28 high-intensity group vs. low-intensity group. GSEA was used to determine whether the functions related to the hub genes with common trend differential expression were enriched at the top (indicating an upregulation trend) or bottom (indicating a downregulation trend) of each group’s ranking. In the results of GSEA, *P*-value < 0.05 was considered statistically significant.

### 2.6 Validation of DEGs through real-time quantitative PCR analysis

To verify the results of RNA-seq, eleven genes were randomly chosen from the identified hub genes in the day 28 low-intensity flea parasitism group vs. control group. Subsequently, the expression levels of these genes were validated using the RT-qPCR method to evaluate the reliability of transcriptome data. The reagent kit method was used to reverse transcribe RNA into cDNA. The RT-qPCR system (20 μL) was as follows: 2 × Taq Pro Universal SYBR qPCR Master Mix, 10 μL; upstream and downstream primers (10 μM), 0.4μL; ddH2O, 7.2 μL and cDNA template, 2 μL. The reaction conditions were as follows: 95°C for 30 s, followed by 40 cycles of 95°C for 10 s and 60°C for 30 s; the collection of melting curve was used as the default process of the instrument. 2^-ΔΔCt^ method was used to determine the relative expression level of the selected gene. HRPT served as an internal reference gene. Each sample was tested three times. The gene information for real-time PCR is given in **Supplementary Table S1**.

### 2.7 Enzyme-linked immunosorbent assay

The concentrations of reactive oxygen species (ROS), superoxide dismutase (SOD), glutathione peroxidase (GSH-Px) and catalase (CAT) were determined using specific ELISA detection kits for hamsters: the ROS cluster ELISA kit, SOD ELISA kit, GSH-Px ELISA kit and CAT ELISA kit, respectively. All reagents were purchased from Shanghai Yuanju Biotechnology Center, with project numbers YJ551049, YJ510872, YJ510877 and YJ510876. The ELISA were conducted in strict accordance with the manufacturers’ protocols. Each sample was executed in triplicate to ensure reliability and reproducibility of the results.

## 3 Results

### 3.1 Weight changes and influencing factors in striped hamsters

**Figure 1:**
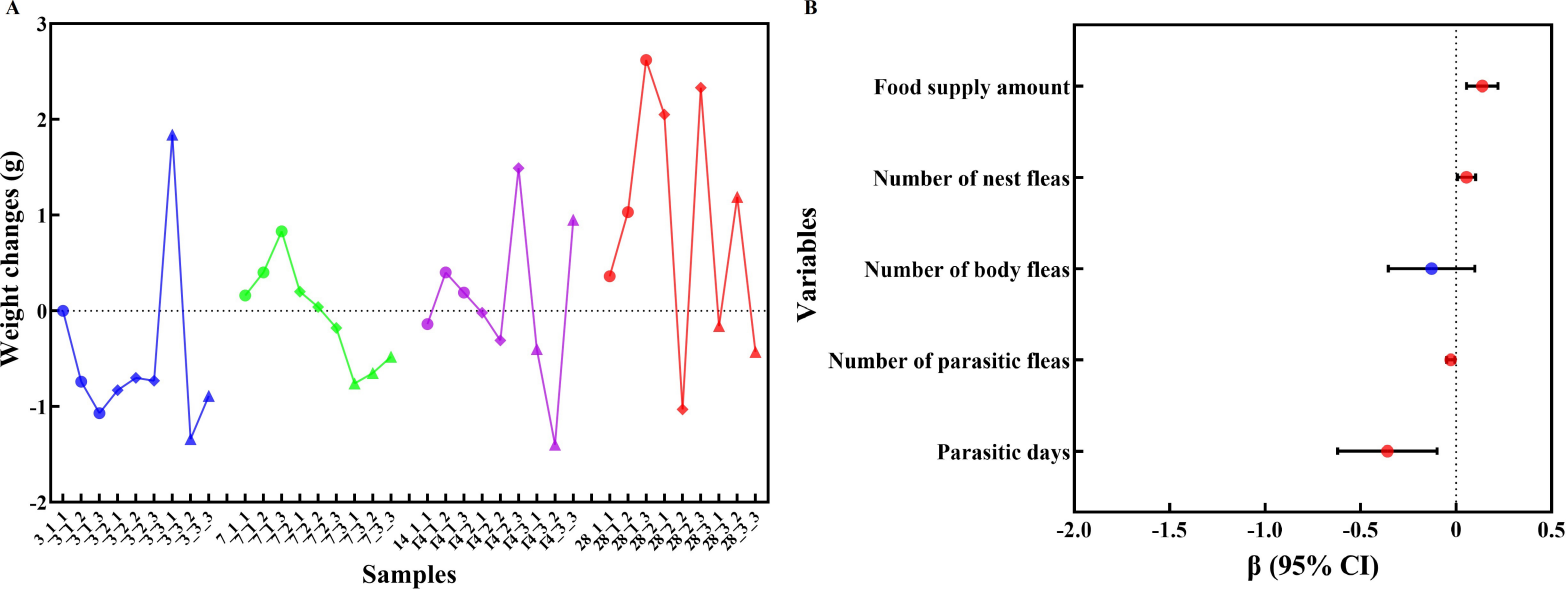
Weight changes and influencing factors of striped hamsters in each group. **(A)** represents the weight changes of striped hamsters in the control group, low-intensity group and high-intensity group after 3, 7, 14 and 28 days of the experiment. On the horizontal axis, the first digit of each number represents the number of days, the second digit represents the group (1 represents the control group, 2 represents the low-intensity group, 3 represents the high-intensity group), and the third digit represents the hamster number within the group. **(B)** represents the influence of parasitic days, number of parasitic fleas, number of body fleas, number of nest fleas and food supply amount on the weight changes in hamsters, respectively. The variables with red dots are the influencing variables with *P* < 0.05.

On day 3, striped hamsters experienced a weight change of -1.34 to 1.84 g. The average weight changes for the control group, low-intensity group and high-intensity group were -0.603, -0.753 and -0.130 g, respectively. By day 7, weight changes ranged from -0.76 to 0.83 g, with averages of 0.463, 0.020 and -0.630 g for the three groups. On day 14, the range was -1.40 to 1.49 g, and the averages were 0.150, 0.387 and -0.283 g. Finally, on day 28, the weight change ranged from -1.03 to 2.62 g, with average changes of 1.337, 1.117 and 0.200 g for the control, low-intensity and high-intensity groups, respectively (**Figure 1**).

A generalized linear regression model was used to explore the relationship between the weight changes of the striped hamster and potential influence factors including parasitism days, flea parasitism intensity, number of fleas on host body, number of fleas found in host nests and weight of intake food. The results showed that there were significant correlations between the parasitic days (β=-0.360, *P* < 0.05), number of parasitic fleas (β=-0.028, *P* < 0.05), number of nest fleas (β=0.054, *P* < 0.05), food supply amount (β=0.137, *P* < 0.05) and weight changes (the difference of weight between the beginning of the experiment and just before euthanasia in hamsters) in striped hamsters (**Figure 1**).

### 3.2 Evaluation of transcriptome sequencing data

The data obtained from sequencing was above 6.0Gb for each sample. The quality scores of Q20 were higher than 96.79% for all samples and Q30 were higher than 91.63%. The GC content ranged between 37.53% and 44.97%. Approximately 88.44% to 95.33% of the clean reads were mapped to the reference genome of *Cricetulus griseus* (ncbi_cricetulus_griseus_gcf_000223135_1_crigri_1_0), including 84.62% to 93.74% uniquely mapped reads. These results indicated that the sequencing data obtained in this study was relatively reliable and could be used for subsequent experimental analysis. The summary of quality control for each sample was given in **Supplementary Table S2**.

### 3.3 Differential gene expression analysis

**Figure 2:**
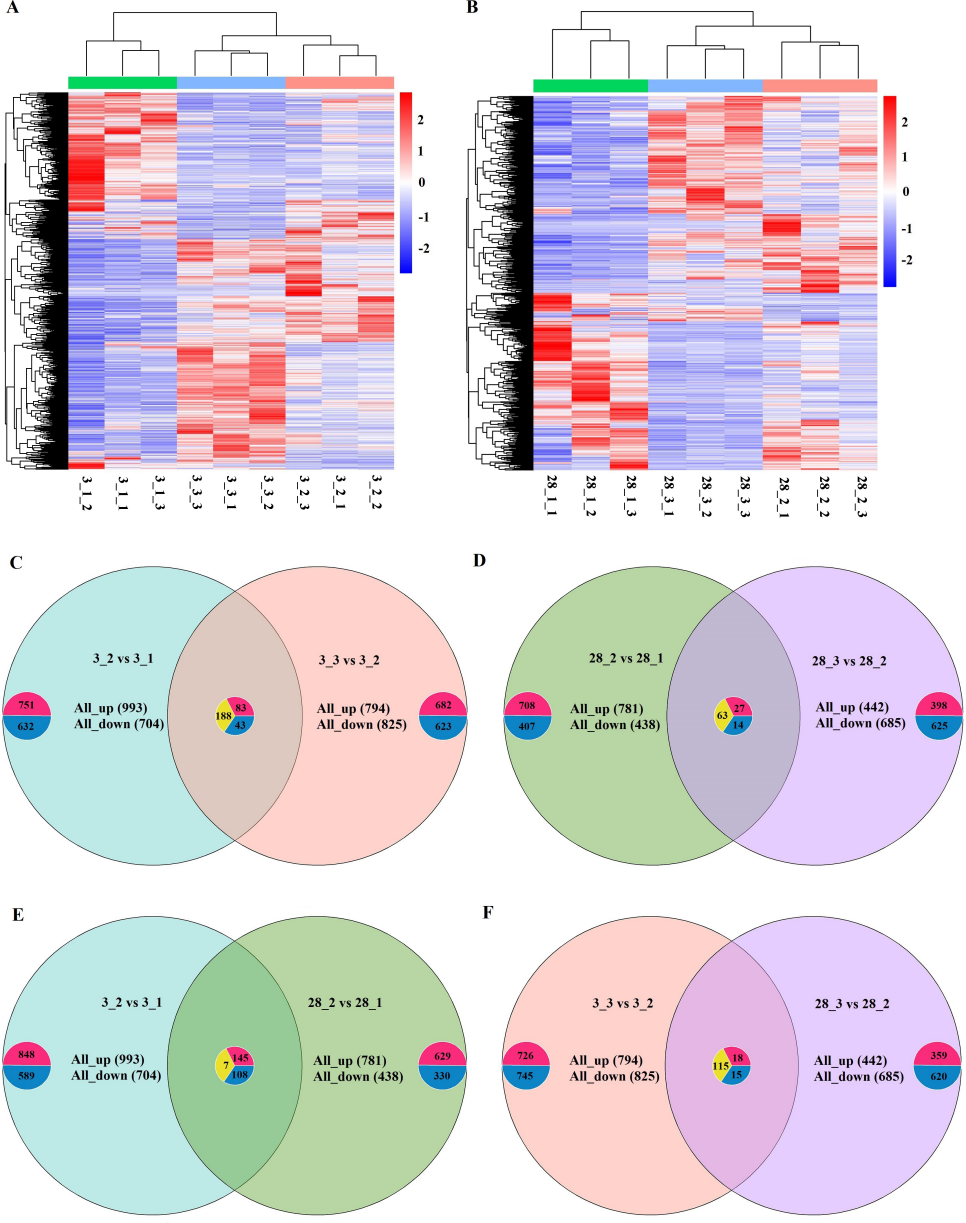
Venn diagrams and heat maps of DEGs in striped hamsters with different flea parasitism intensities during different time periods. **(A, B)** Heat maps of DEGs in striped hamsters with different flea parasitism intensities on the third and twenty-eighth days. The X-axis in the figure represents the sample name, and the Y-axis represents the normalized values of the FPKM of DEGs. Red represents upregulated genes, while blue represents downregulated genes. **(C-F)** The Venn diagrams illustrating the overlapping genes in every two comparison groups. 3_1 represents the control group on the third day. 3_2 represents the low-intensity flea parasitism group on the third day. 3_3 represents the high-intensity flea parasitism group on the third day. 28_1 represents the control group on the 28th day. 28_2 represents the low-intensity flea parasitism group on the 28th day. 28_3 represents the high-intensity flea parasitism group on the 28th day.

The DEGs of striped hamsters parasitized by fleas at different intensities on days 3 and 28 were analyzed. On day 3, compared to the control group, the low-intensity group had 993 genes upregulated and 704 genes downregulated; compared to the low-intensity group, the high-intensity group had 794 genes upregulated and 825 genes downregulated. On day 28, compared to the control group, 781 genes were upregulated and 438 genes were downregulated in the low-intensity group; compared to the low-intensity group, the high-intensity group had 442 genes upregulated and 685 genes downregulated. The expression patterns of significant DEGs in the hamsters’ livers in different time groups were analyzed by hierarchical clustering. The results showed that the expression patterns of flea parasitism were different in different intensity groups, and each group’s samples were clustered according to the group, mainly manifested as three clusters (**Figure 2**).

We summarized the DEGs exhibiting consistent trends across varying intensities of flea parasitism or different time periods. In the early-stage intensity-dependent comparison group, 314 DEGs were shared 83 were consistently upregulated, 43 were uniformly downregulated and 188 displayed contrasting expression patterns. Notably, this number of common trend DEGs diminished in the late-stage intensity-dependent comparison group, comprising 104 genes in total, with 27 upregulated, 14 downregulated and 63 showing opposing expression profiles. In the low-intensity time-dependent comparison group, the scenario was marked by 260 shared DEGs, among which 145 were commonly upregulated, 108 were universally downregulated and a mere 7 exhibited divergent expression tendencies. Conversely, the high-intensity time-dependent comparison group witnessed a substantial reduction in the number of common trend DEGs, amounting to just 18 upregulated, 15 downregulated and 115 with inverse expression patterns (**Figure 2**).

### 3.4 GO enrichment analysis of common trend DEGs

**Figure 3:**
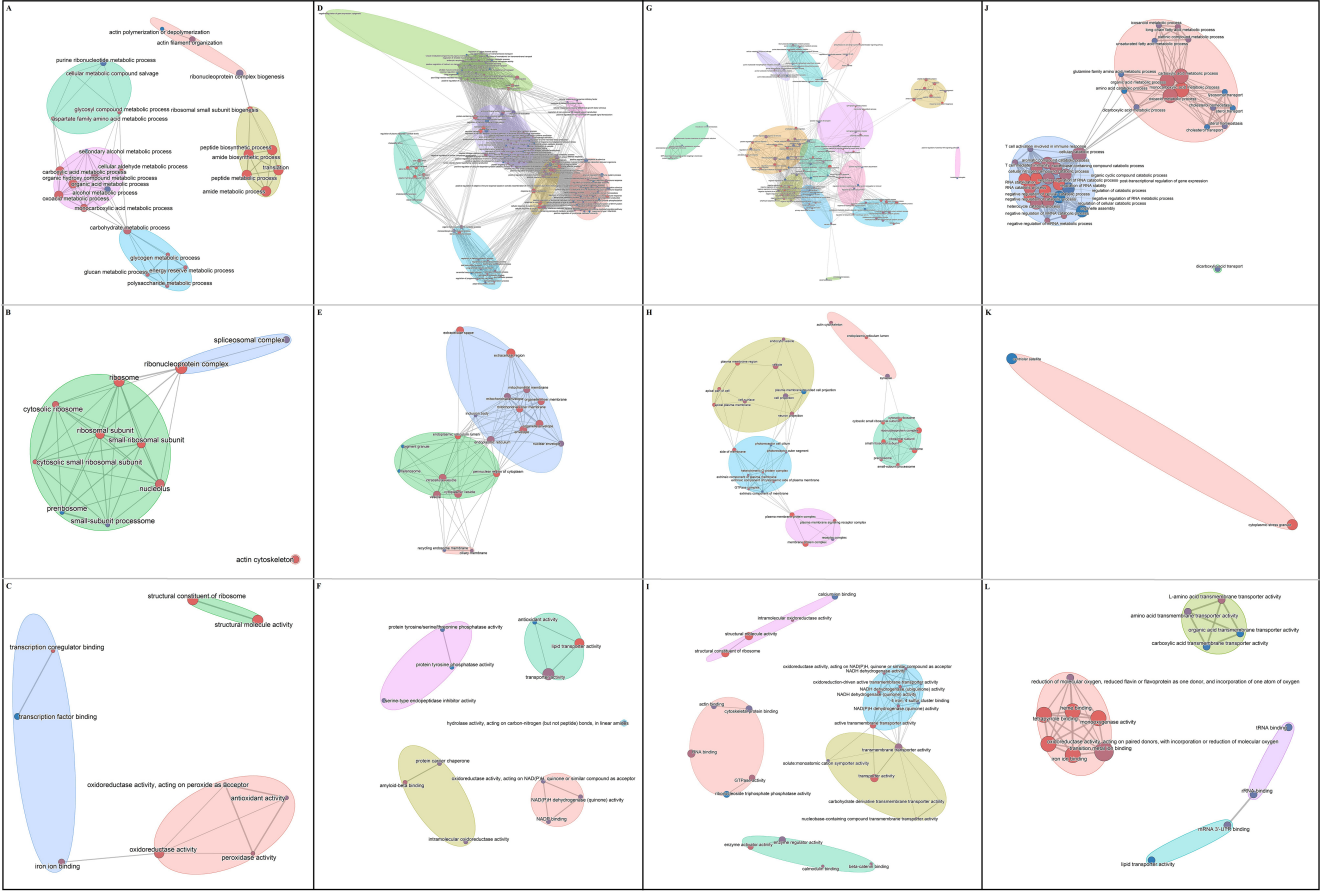
Network Diagram of GO terms enrichment analysis of DEGs. **(A-C)** Biological processes (BP), cellular components (CC) and molecular function (MF) of early-stage intensity-dependent comparison group. **(D-F)** BP, CC and MF of late-stage intensity-dependent comparison group. **(G-I)** BP, CC and MF of low-intensity time-dependent comparison group. **(J-L)** BP, CC and MF of high-intensity time-dependent comparison group.

GO enrichment analysis was performed on the common trend DEGs of the early-stage intensity-dependent comparison group, late-stage intensity-dependent comparison group, low-intensity time-dependent comparison group and high-intensity time-dependent comparison group (**Figure 3**). The number of significantly enriched GO terms in these four groups was 46, 174, 169 and 55, respectively. Multiple GO terms enriched in biological processes were related to host metabolism and immune function. Therefore, we further summarized and analyzed the biological processes.

The common trend of DEGs in the early-stage intensity-dependent comparison group was significantly enriched in 88% (23/26) of GO terms related to metabolic processes, including nucleotide and amino acid metabolism, organic matter metabolism, carbohydrate and energy metabolism, protein synthesis and metabolism. The common trend of DEGs in the late-stage intensity-dependent comparison group showed significant enrichment in 48% (68/141) of GO terms related to metabolic processes, such as lipid metabolism, steroid metabolism and programmed cell death, and lysosomal transport and protein metabolism, and 34% (48/141) terms related to immune processes, including cellular response and signal transduction, immune activation, and immune defense. The common trend of DEGs in the low-intensity time-dependent comparison group had 28% (31/111) of GO terms related to metabolic processes, like nucleotide metabolism, energy metabolism and rhythm regulation, steroid homeostasis, protein synthesis and metabolism, carbohydrate transport and lipid metabolism, and 52% (58/111) terms related to immune processes, involving protein degradation and stress response, macrophage activation and inflammatory response, immunoregulation and intercellular interactions, cell migration and phagocytosis, and immune effects and cytokine response. The common trend of DEGs in the high-intensity time-dependent comparison group was significantly enriched in 45% (17/38) of GO terms related to metabolic processes, including fatty acid and amino acid metabolism and dicarboxylicacid transport, and 55% (21/38) terms related to immune processes, specifically immune response and RNA regulation. A total of 75 DEGs were involved in these metabolic and immune processes (**Supplementary Table S3**).

### 3.5 KEGG enrichment analysis of common trend DEGs

**Figure 4:**
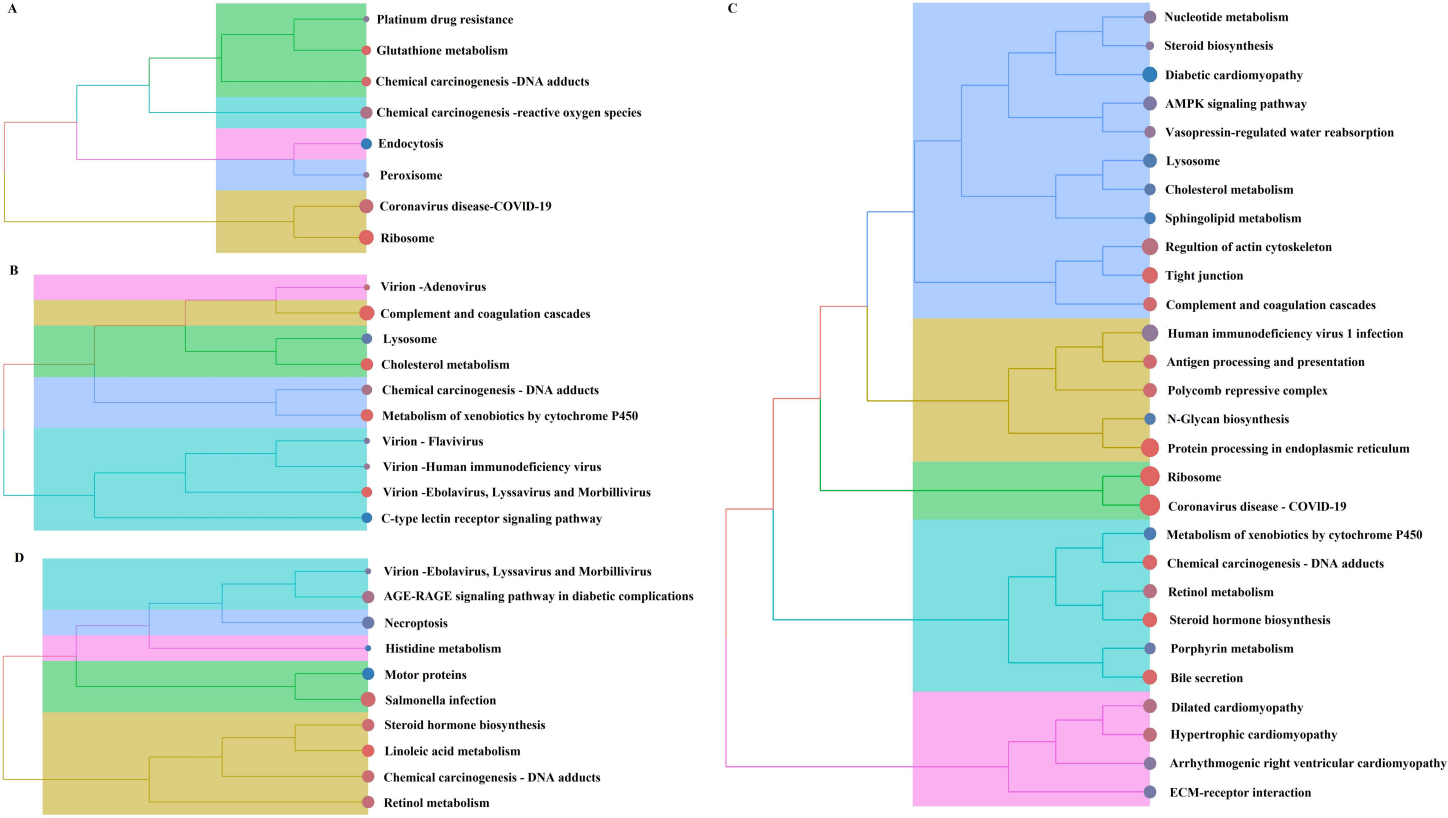
Cluster tree diagram of KEGG pathway enrichment analysis of DEGs. **(A)** Early-stage intensity-dependent comparison group. **(B)** Late-stage intensity-dependent comparison group. **(C)** Low-intensity time-dependent comparison group. **(D)** High-intensity time-dependent comparison group.

We performed KEGG enrichment analysis on the four groups of DEGs exhibiting common trends (**Figure 4**). We found 8, 10, 28 and 10 significantly enriched KEGG pathways in the four groups, all of which were related to host metabolism and immune function. In particular, the chemical carcinogenesis - DNA adducts pathway was commonly involved. There was a total of 129 DEGs involved in these significantly enriched KEGG pathways (**Supplementary Table S4**).

### 3.6 Identification of the hub genes

**Figure 5:**
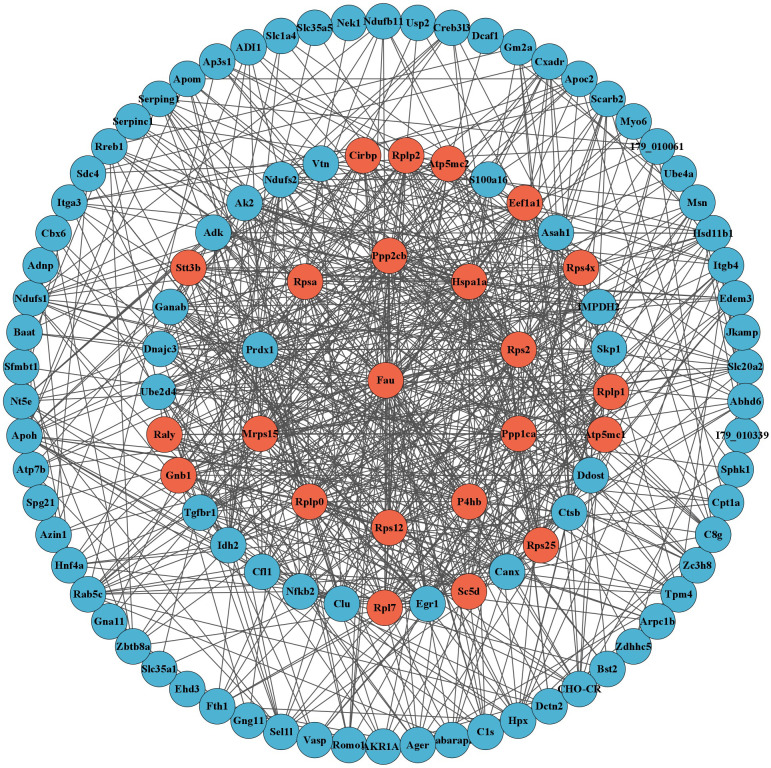
PPI network analysis displayed 110 DEGs related to metabolism and immunity after hiding disconnected nodes in the network. The red circles represent the 23 hub genes obtained through MCODE analysis.

A total of 163 DEGs related to host metabolism and immune function were identified through GO and KEGG enrichment analysis (after removing duplicate DEGs). These DEGs were analyzed by PPI network, and the MCODE plugin was used to extract tightly connected gene cluster modules, resulting in a total of 23 hub genes. The hub genes included Atp5mc1, Atp5mc2, Cirbp, Eef1a1, Fau, Gnb1, Hspa1a, Mrps15, P4hb, Ppp1ca, Ppp2cb, Raly, Rpl7, Rplp0, Rplp1, Rplp2, Rps12, Rps2, Rps25, Rps4x, Rpsa, Sc5d and Stt3b (**Figure 5**).

### 3.7 GSEA related to hub genes

**Figure 6:**
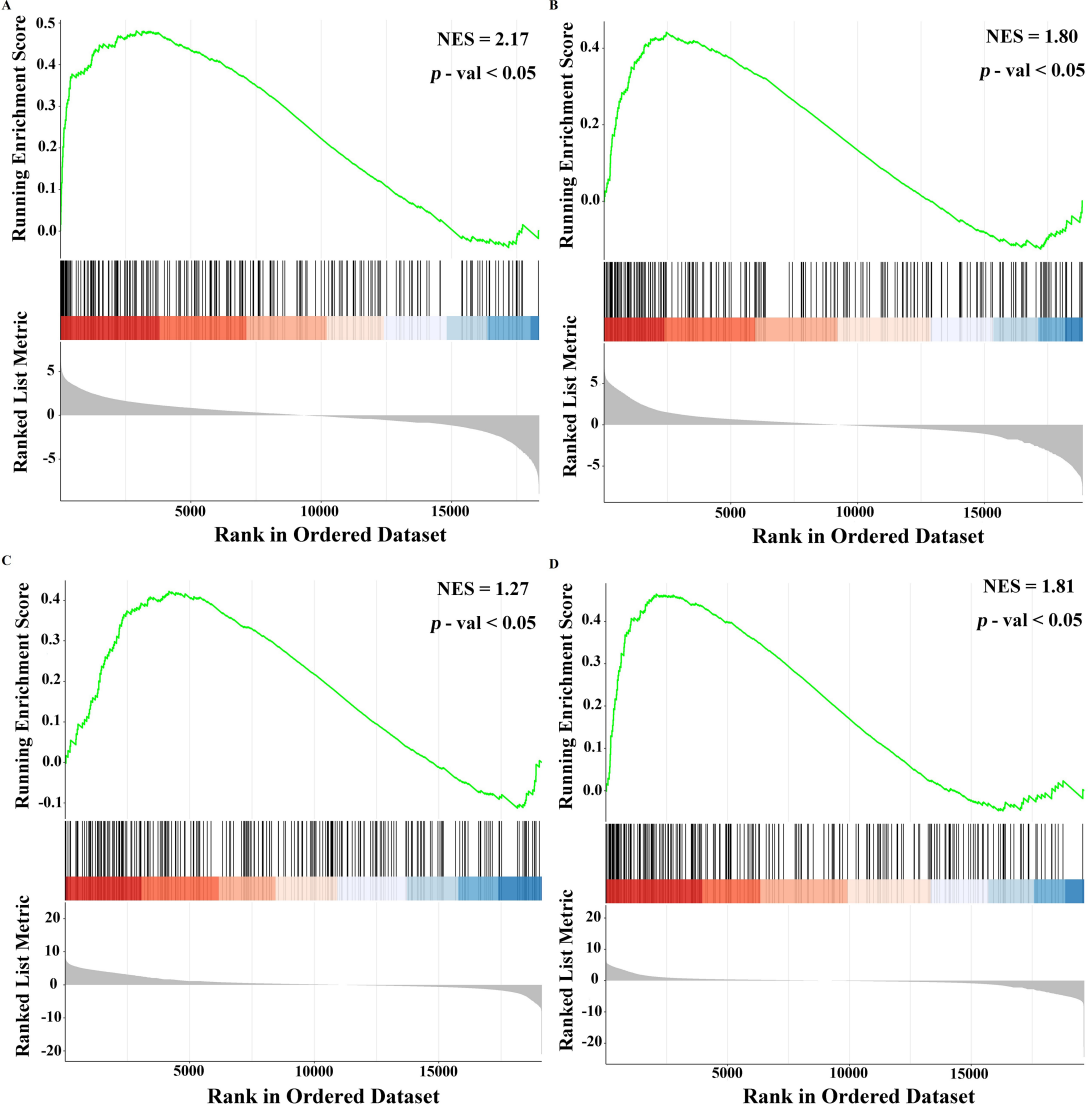
GSEA related to hub genes. 3 days of low-intensity group vs. control group. **(B)** 3 days of high-intensity group vs. low-intensity group. **(C)** 28 days low-intensity group vs. control group. **(D)** 28 days high-intensity group vs. low-intensity group.

To further determine the functions and roles of these hub genes in the host after flea parasitism, we first explored the activity levels of genes related to their functions or pathways through GESA, including the 3 days low-intensity group vs. control group, the 3 days high-intensity group vs. low-intensity group, the 28 days low-intensity group vs. control group and the 28 days high-intensity group vs. low-intensity group. The results showed that these annotated genes in four groups had 98, 67, 35 and 30 significant enrichment pathways, respectively (**Supplementary Table S5**). Among them, three significant enrichment pathways were present in all four groups, namely chemical carcinogenesis - reactive oxygen species, retinol metabolism and chemical carcinogenesis - DNA adducts. The chemical carcinogenesis - reactive oxygen species pathway involved two hub genes, ATP5MC1 and ATP5MC2 (**Figure 6**).

### 3.8 Pathways related to chemical carcinogenesis - reactive oxygen species

**Figure 7:**
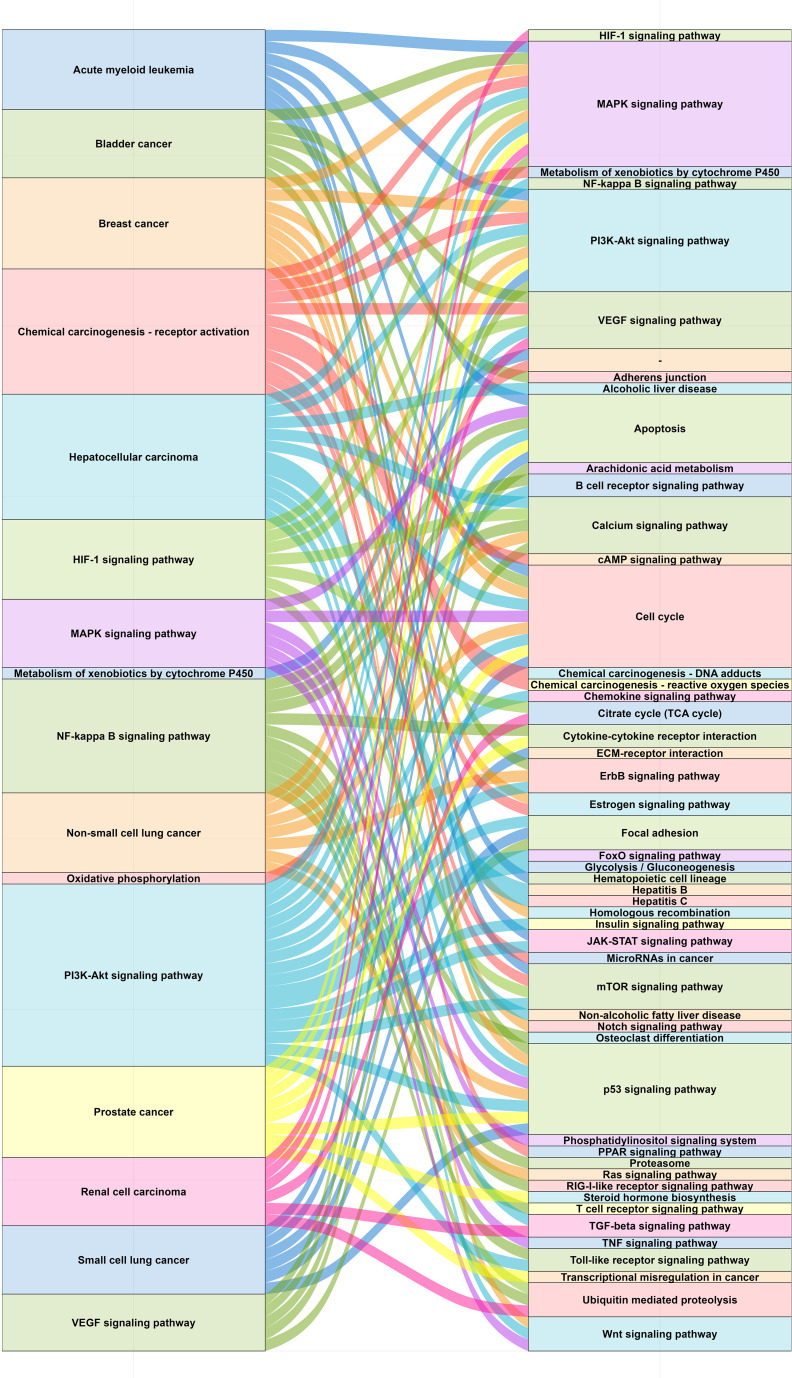
Pathways related to chemical carcinogenesis - reactive oxygen species.

By querying in KEGG (https://www.kegg.jp/kegg/pathway.html), there were 16 pathways associated with chemical carcinogenesis - reactive oxygen species (**Figure 7**). Overall, these pathways were related to the host’s metabolism and immune function. After GSEA, 7 pathways including oxidative phosphorylation, metabolism of xenobiotics by cytochrome P450, MAPK signaling pathway, NF-kappa B signaling pathway, HIF-1 signaling pathway, chemical carcinogenesis - receptor activation and hepatocellular carcinoma showed significant enrichment in some of the four groups: 3 days low-intensity group vs. control group, 3 days high-intensity group vs. low-intensity group, 28 days low-intensity group vs. control group and 28 days high-intensity group vs. low-intensity group (**Supplementary Table S5**). Chemical carcinogenesis - reactive oxygen species was a key pathway connecting these pathways, which not only affected tumor metabolism by disrupting redox signaling but also regulated the activity of multiple signaling pathways. This regulatory effect may lead to oxidative damage and even cell death. However, it was worth noting that moderate levels of reactive oxygen species can act as messengers, participating in the regulation of multiple signaling pathways, suggesting its dual role in cellular physiology and pathology.

### 3.9 Validation of key DEGs by RT-qPCR analysis

**Figure 8:**
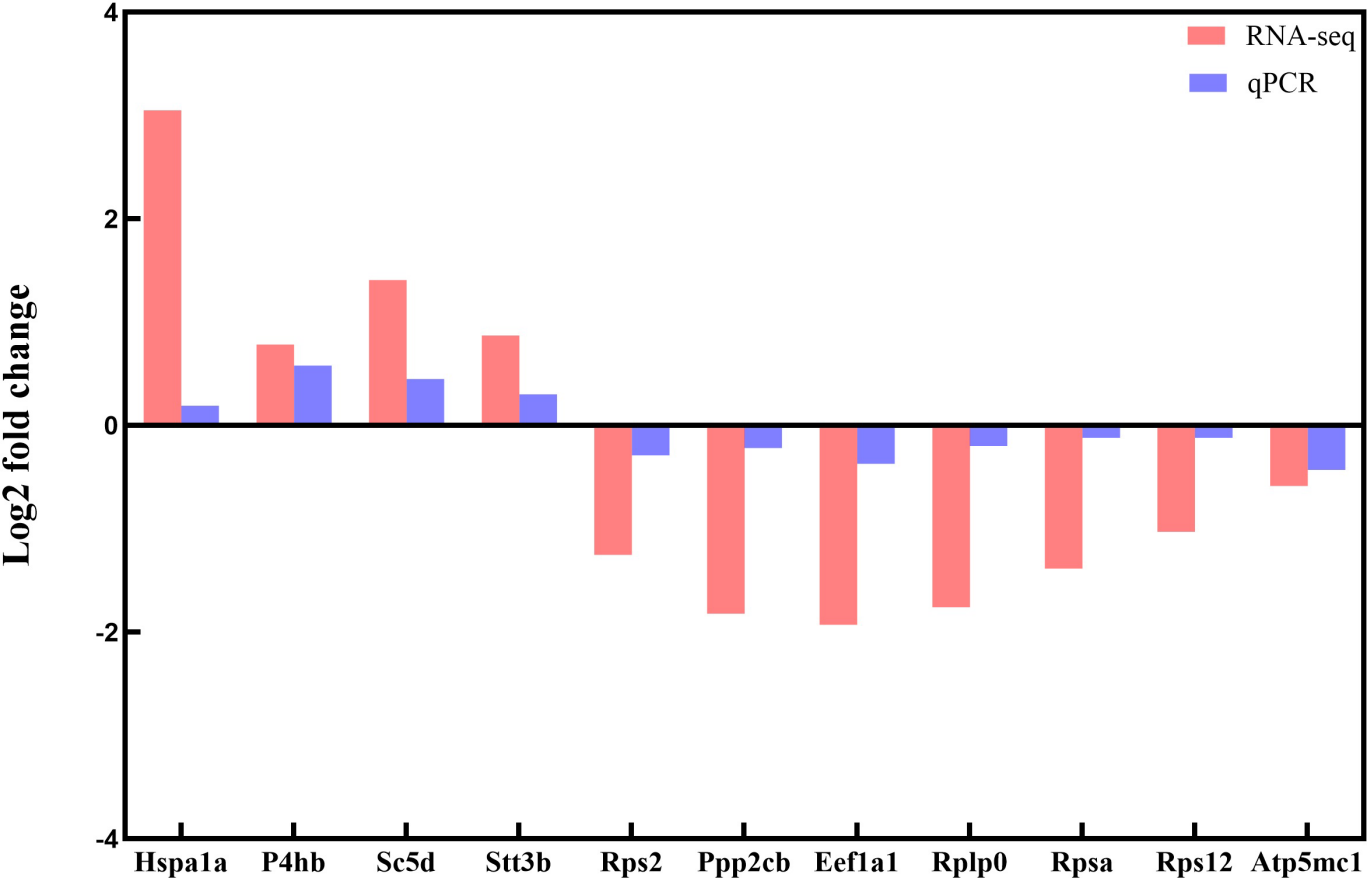
Validation of DEGs between the low-intensity flea parasitism group and the control group after 28 days by RT-qPCR. The x-axis represents the DEGs name, and the y-axis represents the log2 fold change.

To verify the RNA-seq expression results, we used RT-qPCR to evaluate the expression of randomly selected hub DEGs obtained in PPI network analysis, including Hspa1a, P4hb, Sc5d, Stt3b, Rps2, Ppp2cb, Eef1a1, Rplp0, Rpsa, Rps12 and Atp5mc1. The results were consistent with those reflected by RNA-seq (**Figure 8**), indicating the reliability of RNA-seq results.

### 3.10 The levels of ROS, SOD, GSH-Px and CAT were measured by ELISA

**Figure 9:**
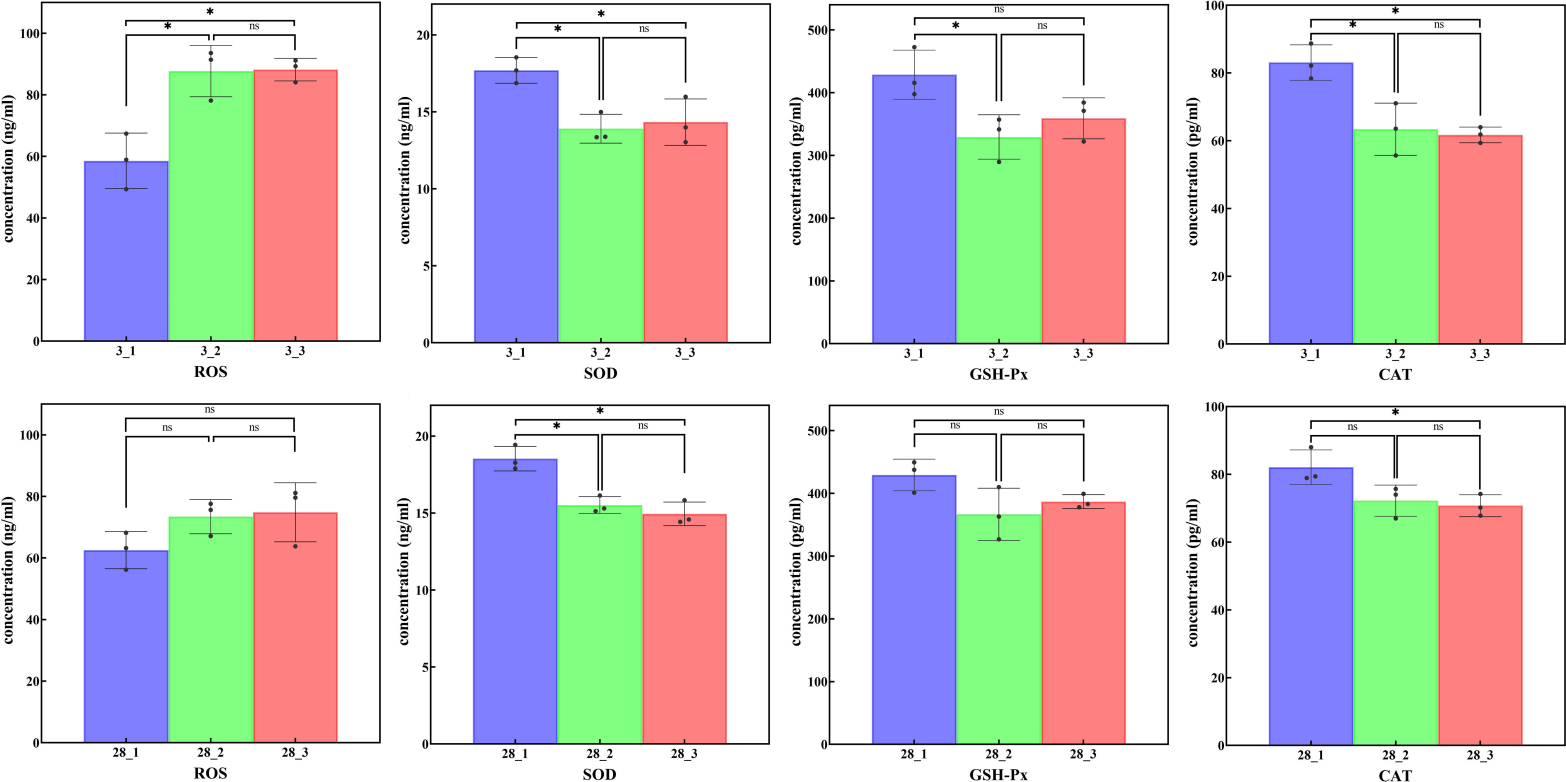
The concentrations of ROS, SOD, GSH-Px and CAT analyzed by ELISA assay *P < 0.05; ns, no significance.

ELISA was employed to assess the protein expression levels of ROS, SOD, GSH-Px and CAT across different groups (**Figure 9**). The findings revealed that on day 3, in contrast to the blank control group, both the low-intensity flea parasitism group and the high-intensity flea parasitism group exhibited a marked elevation in ROS expression. Concurrently, there were significant reductions in the expression levels of SOD and CAT within these two groups. By day 28, when compared to the blank control group, only SOD expression demonstrated notable alterations, specifically displaying a downward trend in both the low-intensity and high-intensity flea parasitism groups.

## 4 Discussion

Previous studies have revealed that ectoparasites parasitism can trigger a series of immune responses in the host (11, 20–22). To gain a deeper understanding of these responses, this study focused on the molecular responses of striped hamsters after experiencing short and long periods of parasitism by *X. cheopis*, offering novel insights into the host’s immune response against ectoparasites. By meticulously examining DEGs under different parasitic intensities and durations, we found significant variations in gene expression tied to metabolic regulation and immune response, illustrating the sophisticated physiological and immunological adjustments hosts undergo when challenged by ectoparasites. Notably, the initial immune response caused by short-term parasitism and the chronic immune response caused by long-term parasitism may have differences in mechanism and intensity, which are reflected through dynamic changes in gene expression.

By exposing hamsters to *X. cheopis* for durations of 3, 7, 14 and 28 days, we found a significant weight decrease with increasing parasitic intensity and duration. This change is likely related to the increased energy consumption of the host in response to ectoparasites parasitism (35). Ectoparasites obtain nutrients from their hosts’ blood to sustain their life (36, 37). Long-term blood-sucking behavior can lead to a significant deficit of nutrients for the host, resulting in weight loss. Moreover, the presence of ectoparasites may also trigger stress responses in the host, which in turn may lead to metabolic disorders and weight changes. To cope with ectoparasites, the host may need to allocate more energy to support immune responses and tissue repair processes, which may sacrifice the energy supply required for other physiological activities (38). These findings align with early research on host-parasite interactions (39–41) that parasitic infections decelerate host growth rates.

GO enrichment analysis showed that in terms of biological processes function, the common trend DEGs in the early-stage intensity-dependent comparison group were significantly enriched in pathways related to the synthesis and metabolism of amino acids, proteins, carbohydrates and organic compounds. This suggests a rapid activation of energy metabolism and resource reallocation by the host to generate sufficient energy and biomolecules to cope with the additional burden caused by ectoparasites, thereby supporting the upcoming immune response. In contrast, the late-stage intensity-dependent comparison group’s common trend DEGs showed significant enrichment in multiple pathways related to immune response. This may reflect the host’s attempt to adjust its metabolic status in response to the infestation of ectoparasites. As the parasitic time extended to the 28th day, most GO terms were enriched in the immune pathway, indicating that the host’s immune system had completed recognition of the ectoparasites and began actively mobilizing immune mechanisms to fight against it. The immune response at this stage includes a series of complex immune regulatory processes such as lymphocyte activation, antibody production and inflammatory response. The transition from short-term metabolic adjustments to long-term immune responses reflects the complex and intricate regulatory mechanisms within the host. KEGG pathway enrichment analysis further revealed the sustained interaction between the host’s metabolic and immune functions during parasitism by ectoparasites. The common trend DEGs of the four groups were significantly enriched in pathways related to metabolic and immune functions, suggesting that the overall physiological state of the host parasitized by fleas is a dynamic equilibrium process involving continuous changes in metabolic regulation and immune response.

The gene expression analysis revealed a significant upregulation of the “chemical carcinogenesis - reactive oxygen species” pathway under varying intensities and durations of parasitism, with hub genes ATP5MC1 and ATP5MC2 being key players. This upregulation is usually associated with oxidative stress, which refers to the imbalance between prooxidants and antioxidants in the body (42, 43). This imbalance can cause cell damage, protein denaturation and DNA mutations, thereby increasing the risk of direct damage to the host’s cells and tissues (44, 45) Reactive oxygen species is crucial in cellular metabolism and inflammatory signaling (46). However, excessive production of reactive oxygen species can cause oxidative damage to cells and tissues (47, 48), activating pathways related to chemical carcinogenesis, including the one mentioned above. The involvement of ATP5MC1 and ATP5MC2 emphasizes their role in regulating the host’s response to this stress by encoding proteins vital for mitochondrial ATP synthesis and electron transport chain function (49–51). Their downregulation may be indicative of impaired mitochondrial function, leading to reduce ATP synthesis and affecting cellular energy supply, reflecting an attempt by the host to mitigate further oxidative damage by decreasing mitochondrial burden. Reactive oxygen species is also an important weapon used by immune cells such as macrophages and dendritic cells to resist pathogens (52–54). Therefore, the upregulation of this pathway signifies the host’s active metabolic regulation to cope with increased energy demands and oxidative damage while mounting an immune response. This regulatory process may exacerbate the host’s weight loss. During the early stages of flea parasitism (3 days), the host likely experiences acute oxidative stress and immune activation. By day 28, as parasitism continues, this reaction may gradually become chronic, involving more biological processes related to long-term stress and inflammation. The continuous involvement of hub genes indicates that the host is striving to maintain physiological homeostasis throughout the entire parasitic process to prevent irreversible damage to the body caused by long-term oxidative damage and inflammatory reactions.

The ELISA results underscored the profound impact of flea parasitism on the host’s oxidative stress response, especially its inhibitory effect on antioxidant enzymes. These findings provide crucial experimental evidence for comprehending the oxidative status within host organisms and the pathological changes potentially induced by parasitic infections. On day 3, both the low-intensity and high-intensity flea parasitism groups exhibited a significant surge in ROS expression levels, whereas the expression levels of SOD and CAT notably diminished. This suggests that flea parasitism may trigger oxidative stress responses in hosts, consistent with existing literature (55, 56). The heightened oxidative stress could be attributed to the parasites causing hamsters to produce higher levels of free radicals, leading to cellular and tissue damage. SOD, an important antioxidant enzyme, converts superoxide anions into hydrogen peroxide, while CAT further converts into water and oxygen (57). The decrease in SOD and CAT expression levels signifies potential impairment or inhibition of the body’s antioxidant defense mechanisms, exacerbating oxidative stress levels. By day 28, despite no significant alteration in ROS levels, there was a marked reduction in SOD expression in both parasitism groups. This result reflects the sustained suppression of antioxidant enzyme activity caused by long-term flea parasitism, possibly related to the body’s prolonged exposure to an oxidative stress environment. Long-term oxidative damage may lead to a further reduction in intracellular antioxidant enzyme expression, establishing a deleterious feedback loop.

In this study, we selected liver tissues to study the host’s molecular response to flea parasitism. This choice was based on several considerations. Firstly, the liver plays a vital role in systemic immune responses and is an important organ for regulating inflammation and immune reactions (58–60). We believe it can provide significant insights into the immune response to ectoparasitic challenges. Secondly, through long-term observation of hamster body weight, we found a significant weight loss in parasitized animals. Given that the liver is a crucial metabolic organ (61), the liver transcriptome can reveal deeper insights into the potential mechanisms behind the host’s weight loss. Additionally, existing studies (62, 63)indicate that liver transcriptomic analysis can unveil changes occurring in the host during immune responses and metabolic processes. However, different tissues may have varying responses, and future research should consider using multiple tissues for a comprehensive analysis. For example, simultaneous study of tissues such as skin, blood and spleen may provide a more holistic understanding of the overall impact of ectoparasitic infections on the host. Furthermore, we have validated the RNA-seq results through RT-qPCR and ELISA methods. However, additional experimental validations can further strengthen the confirmation of the observed gene expression patterns. In future studies, integrating proteomics approaches could provide a more comprehensive analysis and verification of the results presented in this study, as well as offer deeper insights into potential biological processes.

## 5 Conclusion

This study has unveiled the intricate mechanisms of metabolic regulation and immune response in stripped hamsters subjected to flea infestation through comprehensive transcriptomic analysis. The results showed that under different parasitic intensities and durations, hosts exhibited different metabolic adjustments and immune response strategies as revealed by the dynamic changes in gene expression. This study not only deepens our understanding of the host’s metabolic regulation and immune response in the face of ectoparasitic challenges but also enriches our understanding of host-ectoparasite interactions and how hosts coordinate complex biological processes to respond to parasitic challenges.

## Data Availability

The datasets presented in this study can be found in online repositories. The names of the repository/repositories and accession number(s) can be found below: PRJNA1184124 (SRA).
