# Supplementary material for: Metabolism and immune responses of striped hamsters to ectoparasite challenges: insights from transcriptomic analysis
Source: Front Immunol. 2024 Dec 12;15:1516382. doi: 10.3389/fimmu.2024.1516382 (PMC11669363; doi:10.3389/fimmu.2024.1516382)
Supplement: Supplementary file 1 [file DataSheet1.zip › Supplementary materials/Table S1-S2.doc]

**Table S1 Primers for RT-qPCR**

| Name | Forward primer | Reverse primer |
| --- | --- | --- |
| HPRT1 | GAAAGACTTGCCCGAGATGT | AGTCATGGGAATGGATCTAT |
| Hspa1a | GCAGGAGGCCGAGAGGTACA | TCCTGTCGGCCTCGCTGATC |
| P4hb | GGAGGCGAAATCAAGACACA | GGCCAAAGAACTCAAGGATA |
| Sc5d | TTCTCAATGTTGCCGATCAC | GGTTGCACAGAAGAAGTAGA |
| Stt3b | CGATCAGTGCCGTCTACTTT | CATGTCATCTCCCAGATAGT |
| Rps2 | CGTGGAGGTAAAGCCGAAGA | GGGCAGGGAGAACAGGTAGA |
| Ppp2cb | TATCTATTCATGGGTGACTA | TCATCATAAAAGCCATAGAC |
| Eef1a1 | GGATGGAAAGTCACCCGTAA | CGAGTTGGTGGCAGGATACA |
| Rplp0 | TTGCACTCACGCTTCCTAGA | ACCCGCTTGTAACCATTGAT |
| Rpsa | CACGGCTTCTAGTGGTCAC | ACGTAGCGGAGAGGAGAGTC |
| Rps12 | GAGGCCCTTTGTGCTGAACA | CTGAGATTCTTTGCCATAGT |
| Atp5mc1 | AGGCACTGCTCGTTACTC | TGCAGGAAGGCTGTTTAGAT |

**Table S2 Summary of sequence quality and mapped data of samples**

| sample | raw_reads | raw_bases | clean_reads | clean_bases | error rate(%) | Q20(%) | Q30(%) | GC content(%) | Total mapped | Multiple mapped | Uniquely mapped |
| --- | --- | --- | --- | --- | --- | --- | --- | --- | --- | --- | --- |
| H1 | 43567170 | 6.54G | 42355322 | 6.35G | 0.03 | 97.97 | 93.95 | 37.79 | 40378693(95.33%) | 673524(1.59%) | 39705169(93.74%) |
| H2 | 47110364 | 7.07G | 45557898 | 6.83G | 0.02 | 98.12 | 94.25 | 38.04 | 42582978(93.47%) | 867971(1.91%) | 41715007(91.56%) |
| H3 | 45988350 | 6.9G | 43791010 | 6.57G | 0.03 | 97.62 | 93.08 | 37.53 | 41614298(95.03%) | 919177(2.1%) | 40695121(92.93%) |
| H19 | 46082194 | 6.91G | 42396696 | 6.36G | 0.03 | 96.83 | 91.91 | 41.45 | 37748673(89.04%) | 800399(1.89%) | 36948274(87.15%) |
| H20 | 43594004 | 6.54G | 41925308 | 6.29G | 0.03 | 97.76 | 93.91 | 41.54 | 39074057(93.2%) | 740255(1.77%) | 38333802(91.43%) |
| H23 | 41711936 | 6.26G | 40229640 | 6.03G | 0.03 | 96.92 | 91.69 | 38.68 | 37485679(93.18%) | 687822(1.71%) | 36797857(91.47%) |
| H25 | 42055226 | 6.31G | 40239636 | 6.04G | 0.03 | 96.79 | 91.63 | 41.74 | 35586532(88.44%) | 776061(1.93%) | 34810471(86.51%) |
| H28 | 44754700 | 6.71G | 41521170 | 6.23G | 0.03 | 96.94 | 92.07 | 41.96 | 37583238(90.52%) | 801477(1.93%) | 36781761(88.59%) |
| H29 | 46349658 | 6.95G | 44562328 | 6.68G | 0.03 | 96.98 | 91.78 | 38.58 | 42133512(94.55%) | 601787(1.35%) | 41531725(93.2%) |
| H4 | 41856474 | 6.28G | 38844766 | 5.83G | 0.03 | 97.28 | 92.35 | 38.37 | 35521546(91.44%) | 794759(2.05%) | 34726787(89.4%) |
| H8 | 40966682 | 6.15G | 39900500 | 5.99G | 0.03 | 96.98 | 91.87 | 40.65 | 36727531(92.05%) | 542509(1.36%) | 36185022(90.69%) |
| H10 | 44550156 | 6.68G | 43126056 | 6.47G | 0.03 | 97.76 | 93.92 | 41.58 | 40092927(92.97%) | 830061(1.92%) | 39262866(91.04%) |
| H13 | 46399498 | 6.96G | 43469004 | 6.52G | 0.03 | 97.24 | 92.38 | 39.21 | 39778974(91.51%) | 1103543(2.54%) | 38675431(88.97%) |
| H16 | 42364980 | 6.35G | 40159378 | 6.02G | 0.03 | 97.68 | 93.37 | 38.48 | 37405780(93.14%) | 840237(2.09%) | 36565543(91.05%) |
| H18 | 43344834 | 6.5G | 41284984 | 6.19G | 0.03 | 97.69 | 93.39 | 38.55 | 38668068(93.66%) | 806842(1.95%) | 37861226(91.71%) |
| H26 | 45386704 | 6.81G | 42069144 | 6.31G | 0.03 | 97.22 | 92.19 | 37.92 | 39360239(93.56%) | 915667(2.18%) | 38444572(91.38%) |
| H31 | 43837144 | 6.58G | 42684582 | 6.4G | 0.03 | 96.87 | 91.78 | 42.91 | 39502852(92.55%) | 729650(1.71%) | 38773202(90.84%) |
| H35 | 42133972 | 6.32G | 41052884 | 6.16G | 0.03 | 97.22 | 92.32 | 39.2 | 36364439(88.58%) | 561697(1.37%) | 35802742(87.21%) |
| H7 | 48350300 | 7.25G | 47685676 | 7.15G | 0.03 | 97.9 | 93.95 | 38.3 | 43484685(91.19%) | 814870(1.71%) | 42669815(89.48%) |
| H11 | 46637620 | 7G | 44881738 | 6.73G | 0.03 | 97.86 | 94.23 | 42.9 | 38707086(86.24%) | 726065(1.62%) | 37981021(84.62%) |
| H12 | 48685532 | 7.3G | 47515430 | 7.13G | 0.02 | 98.04 | 94.53 | 42.98 | 43055798(90.61%) | 881814(1.86%) | 42173984(88.76%) |
| H14 | 41540038 | 6.23G | 40735512 | 6.11G | 0.02 | 98.17 | 94.91 | 44.97 | 37464359(91.97%) | 910795(2.24%) | 36553564(89.73%) |
| H21 | 49728606 | 7.46G | 48465762 | 7.27G | 0.03 | 97.42 | 93.25 | 39.14 | 45323230(93.52%) | 913527(1.88%) | 44409703(91.63%) |
| H22 | 45141260 | 6.77G | 43192796 | 6.48G | 0.03 | 97.32 | 93.39 | 41.35 | 39348800(91.1%) | 1211870(2.81%) | 38136930(88.29%) |
| H27 | 42734256 | 6.41G | 41707404 | 6.26G | 0.02 | 98.27 | 95.09 | 44.08 | 38058025(91.25%) | 868012(2.08%) | 37190013(89.17%) |
| H32 | 42870468 | 6.43G | 41781382 | 6.27G | 0.02 | 98.1 | 94.83 | 44.2 | 38491450(92.13%) | 859817(2.06%) | 37631633(90.07%) |
| H36 | 54651092 | 8.2G | 53524154 | 8.03G | 0.02 | 98.23 | 95 | 44.01 | 49866252(93.17%) | 1063824(1.99%) | 48802428(91.18%) |
